# Hypoxia‐Driven Neurovascular Impairment Underlies Structural‐Functional Dissociation in Diabetic Sudomotor Dysfunction

**DOI:** 10.1002/mco2.70173

**Published:** 2025-04-24

**Authors:** Xu Guo, Chao Zhang, Yuzhen Wang, Zhao Li, Yaxin Tan, Dongzhen Zhu, Wei Song, Yi Kong, Jinpeng Du, Yuyan Huang, Liting Liang, Jianjun Li, Mengde Zhang, Linhao Hou, Qinhua Liu, Feng Tian, Bingyang Yu, Yue Kong, Zhenyu Zhou, Xiaobing Fu, Sha Huang

**Affiliations:** ^1^ College of Graduate Tianjin Medical University Tianjin People's Republic of China; ^2^ Research Center for Tissue Repair and Regeneration Affiliated to the Medical Innovation Research Department PLA General Hospital and PLA Medical College Beijing People's Republic of China; ^3^ Research Unit of Trauma Care, Tissue Repair and Regeneration Chinese Academy of Medical Sciences Beijing People's Republic of China; ^4^ School of Medicine Nankai University Tianjin People's Republic of China; ^5^ Department of Orthopedics The 960th Hospital of the PLA Joint Logistics Support Force Jinan People's Republic of China; ^6^ PLA Key Laboratory of Tissue Repair and Regenerative Medicine and Beijing Key Research Laboratory of Skin Injury Repair and Regeneration Beijing People's Republic of China

**Keywords:** neural support, neurovascular network, sudomotor dysfunction, sweat gland cell–neural cell interaction model, sweat gland microenvironment

## Abstract

Sudomotor dysfunction in diabetic patients increases the risk of fissures, infections, and diabetic foot ulcers (DFUs), thereby reducing the quality of life. Despite its clinical importance, the mechanisms underlying this dysfunction remain inadequately elucidated. This study addresses this gap by demonstrating that despite structural integrity, sweat glands (SGs) in diabetic individuals with DFUs, and a murine model of diabetic neuropathy (DN), exhibit functional impairments, as confirmed by histological and functional assays. Integrated transcriptome and proteome analysis revealed significant upregulation of the SG microenvironment in response to hypoxia, highlighting potential underlying pathways involved. In addition, histological staining and tissue clearing techniques provided evidence of impaired neurovascular networks adjacent to SGs. Single‐cell RNA sequencing unveiled intricate intercellular communication networks among endothelial cells (ECs), neural cells (NCs), and sweat gland cells (SGCs), emphasizing intricate cellular interactions within the SG microenvironment. Furthermore, an in vitro SGC–NC interaction model (SNIM) was employed to validate the supportive role of NCs in regulating SGC functions, highlighting the neurovascular‐SG axis in diabetic pathophysiology. These findings confirm the hypoxia‐driven upregulation of the SG microenvironment and underscore the critical role of the neurovascular‐SG axis in diabetic pathophysiology, providing insights into potential therapeutic targets for managing diabetic complications and improving patient outcomes.

## Graphical abstract

The progressive functional impairment of sweat glands in diabetic sudomotor dysfunction often precedes structural changes. The underlying pathogenesis of the dysfunctional but structurally normal phase of sweat gland dysfunction can be attributed to three main factors: cellular hypoxia, insufficient vascularization, and a reduction in the number of nerves innervating the sweat glands.

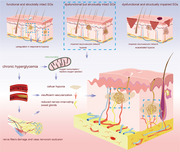

## 1 Introduction

Diabetes mellitus (DM) is a complex metabolic disorder characterized by chronic hyperglycemia resulting from defects in insulin secretion and/or action. It poses a significant global health burden due to its serious and costly nature, impacting multiple organ systems, such as the kidneys, eyes, heart, and brain, and leading to complications such as nephropathy, retinopathy, neuropathy, cardiopathy, and encephalopathy [1]. Amidst these well‐recognized complications, sudomotor dysfunction—a less acknowledged yet clinically relevant issue—presents as reduced sweating and skin dryness, predisposing individuals, especially those with diabetic foot, to ulcerations and infections.

Despite the well‐documented systemic effects of DM on vital organs, its impact on the integumentary system, particularly on SG function, has received less attention despite its significant implications for patient health [2]. Studies have highlighted that individuals with Type 2 DM often exhibit sudomotor dysfunction and structural abnormalities in SGs, including smaller ducts, compared to non‐diabetic individuals [3]. In diabetic patients with angiopathy, inadequate oxygen, and nutrient supplies to secretory cells can lead to SG atrophy [4]. The reciprocal interaction between the vascular niche and SGs plays a crucial role in promoting SG regeneration, highlighting the importance of microvascular network reconstruction for restoring SG function [5, 6]. Insufficient vascularization directly impairs both the physiological function and anatomical structure of SGs, underscoring the intricate interplay between vascular health and sudomotor dysfunction in DM.

DN stands out as one of the earliest and most significant complications in individuals with DM. Initially, DM triggers reversible microangiopathy, which progresses to irreversible changes due to protein damage of nerve fibers and occlusion of the vasa nervorum. These processes lead to Schwann cell dysfunction and nerve fiber demyelination, culminating in DN [7]. Histopathological examinations in diabetic patients with DN‐associated hypohidrosis and compensatory hyperhidrosis have revealed denervation of eccrine SGs, suggesting that nerve fiber damage is a primary cause of hypohidrosis [8]. Similarly, skin biopsies from patients with Ross syndrome demonstrated reduced cholinergic innervation of SGs in hypohidrotic areas [9]. Studies using diabetic mice have also shown that sudomotor dysfunction is associated with diminished autonomic innervation, indicating a clear link between nerve damage and impaired SG function [10].

Chronic hyperglycemia promotes the production of reactive oxygen species (ROS), leading to oxidative stress that induces endothelial cell proliferation, thickening of the basement membrane, and increased vascular permeability, ultimately causing microvascular complications. This compromised blood supply to the NCs results in axonal degeneration, damage to the myelin sheath, and reduced nerve conduction. When sympathetic postganglionic fibers innervating the SGs are affected, sudomotor dysfunction ensues. Understanding these mechanisms of DM‐induced sudomotor dysfunction is crucial for preventing anatomical and physiological damage to SGs. However, the current understanding of the relationship between neurovascular health and SG function in diabetic patients remains incomplete, necessitating a more comprehensive approach to address this knowledge gap.

This study aims to uncover the intricate interplay between the neurovascular system and SGs in DM, with a specific focus on hypoxia‐induced alterations within the SG microenvironment. Through the integration of multi‐omics approaches, advanced imaging technologies, and in vitro modeling, we aim to elucidate the complex cellular crosstalk and detrimental effects of hypoxia on SG function. Our findings emphasize the critical importance of maintaining a healthy neurovascular‐SG axis and underscore hypoxia as a pivotal mediator in the development of sudomotor dysfunction. This investigation contributes to a more nuanced understanding of diabetic complications and proposes potential therapeutic strategies for preserving SG function and mitigating subsequent complications in diabetic patients.

## 2 Results

### 2.1 Structural‐Functional Dissociation in Diabetic Sudomotor Dysfunction

**FIGURE 1:**
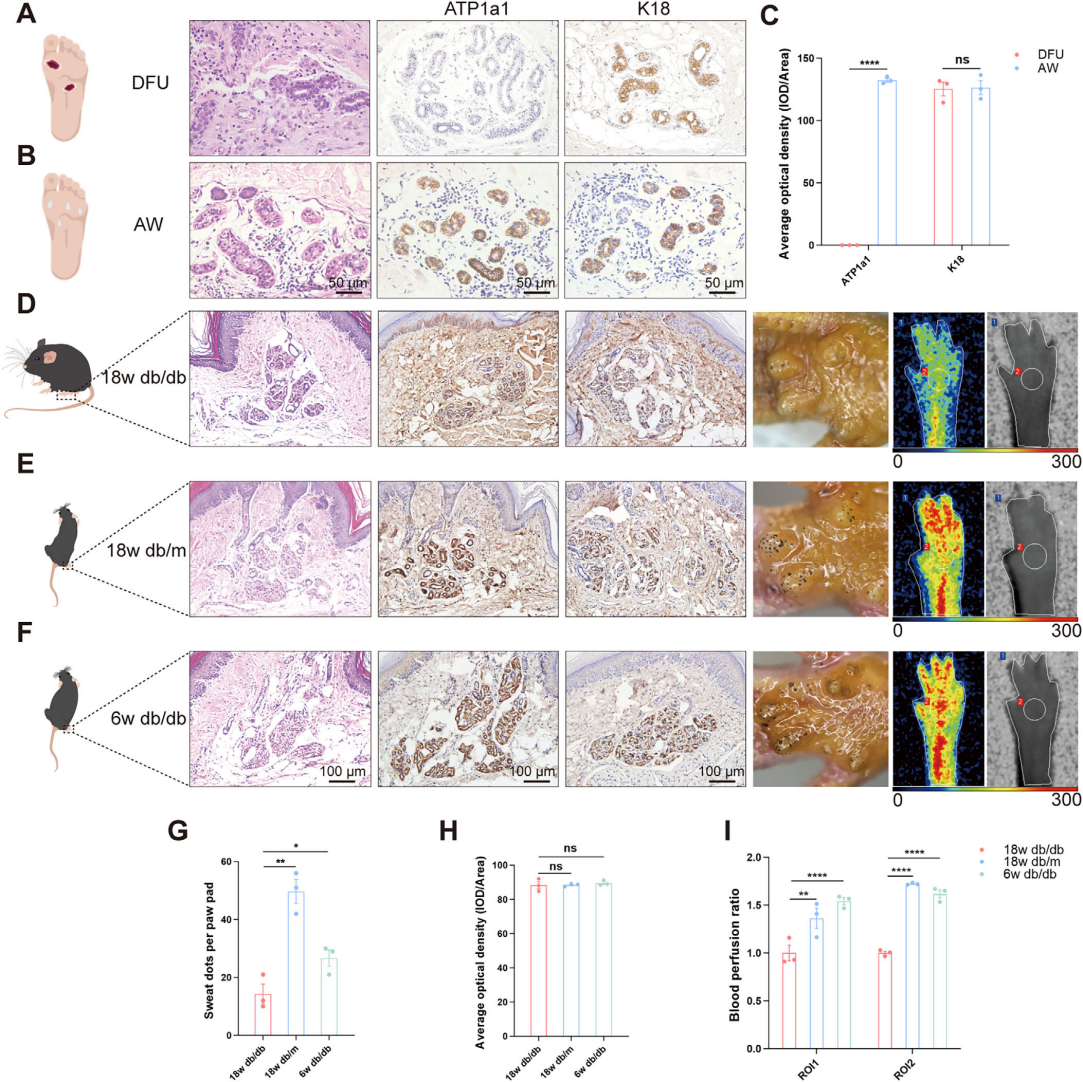
Structurally intact but functionally impaired sweat glands (SGs) in diabetic foot ulcer (DFU) patients and in diabetic neuropathy (DN) model mice. (A, B) Hematoxylin and eosin (HE) staining and immunohistochemical (IHC) staining of ATPase Na+/K+ transporting subunit alpha 1 (ATP1a1, SG functional marker) and cytokeratin 18 (K18, SG luminal cell marker) in skin samples from DFU and AW (acute wound) patients (scale bar: 50 µm). (C) Statistical analysis of immunohistochemical staining of ATP1a1 and K18. *n* = 3 per group. (D–F) Morphological and functional assessment of SGs in 18w db/db, 18w db/m, and 6w db/db mice: H&E staining and IHC staining (left panel), sweat assay (middle panel) and paw perfusion (right panel) (scale bar: 100 µm). (G) Statistical analysis of sweat dots on the paw pads, IHC staining of K18 (H) and blood flow of the region of interests (ROIs) of 18w db/db, 18w db/m, and 6w db/db mice (I). *n* = 3 per group. Each symbol in the statistical graph (G) represents the number of dark dots observed in the sweating experiment for each individual mouse.

To ascertain whether the reduction in sweating is attributable to dysfunction, structural changes, or a depletion in the number of SGs, HE and immunohistochemical staining were used to assess the function, structure, and number of SGs in skin samples from patients with DFU and normal subjects. Our findings revealed that the morphology of ductal branches and gland structure remained intact in both groups and the ATPase Na+/K+ transporting subunit alpha 1 (ATP1a1), a functional marker of SGs, was absent in the DFU group but was abundantly expressed in the acute wound (AW) group. In contrast, cytokeratin 18 (K18), a phenotypic biomarker distributed in SG secretory ducts or the myoepithelium, was not differentially expressed between the aforementioned two groups. Furthermore, there was no notable decrease in the number of SGs, suggesting that the functional integrity of the SGs in the DFU group was impaired but that their structural integrity was maintained (Figure 1A–C).

Given that db/db mice can effectively simulate characteristics of human DN, including progressive sensory function loss and electrophysiological abnormalities, and that the density of intraepidermal nerve fibers in the skin of 18w db/db mice is significantly reduced—closely correlating with sensory loss—this condition is considered an early manifestation of DN [11]. Therefore, this study employs this model to investigate the evolutionary mechanisms underlying sudomotor dysfunction. The SGs in 18w db/db mice were found to be dysfunctional but structurally intact, compared with those in 18w db/m and 6w db/db mice (Figure 1D–H), and the number of SGs was not significantly reduced according to immunohistochemical and HE staining. In addition, sweating experiments were conducted, which revealed that 18w db/db mice exhibited significantly less sweating than the other two groups. In light of the reciprocal interaction between SGs and the vascular niche [5], a significant reduction in overall foot perfusion (region of interest 1 [ROI1]) and perfusion of the SG region (region of interest 2 [ROI2]) was revealed in 18w db/db mice when compared with those in the other two groups by laser Doppler imaging (Figure 1D–F,I), indicating that reduced blood flow was associated with sudomotor dysfunction. This finding is consistent with research showing that impaired circulation can lead to reduced sweat production [12, 13] and reduced vascular network‐related attenuation of SG function [6].

### 2.2 SG Microenvironment, DFU, and DN Were Upregulated in Response to Hypoxia

**FIGURE 2:**
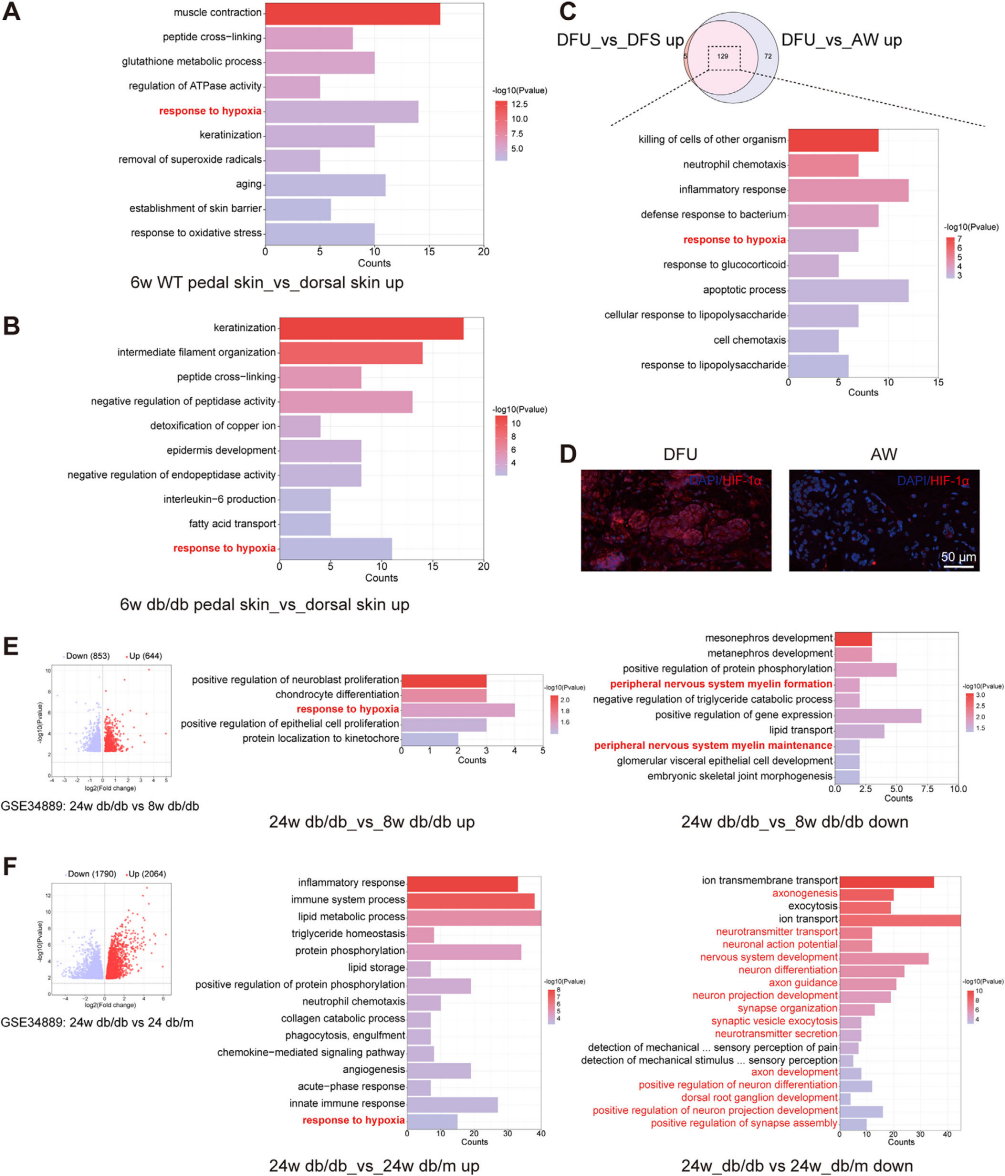
The SG microenvironment, DFU, and DN are upregulated in response to hypoxia. (A, B) Gene ontology (GO) enrichment analysis of the SG microenvironment in 6w wild type (WT) and 6w db/db mice. (C) GO enrichment analysis of the overlapping 129 genes between the DFU_vs_AW up group and the DFU_vs_DFS up group; DFS, diabetic foot skin (D) Immunofluorescence staining for HIF‐1α in skin samples from DFU patients and AW patients (Scale bar: 50 µm). (E, F) Volcano plot (left panel) and GO enrichment analysis of differentially expressed genes (DEGs) between the sciatic nerves of 24w and 8w db/db mice and between 24w db/db and 24w db/m mice. The middle panel indicates upregulated DEG enrichment analyses and the right panel indicates downregulated DEG enrichment analyses.

The exclusive presence of eccrine SGs in the paw pads of mice indicates that the microenvironment in which these glands develop and function is more specific. To elucidate the mechanism of sudomotor dysfunction due to reduced perfusion, proteomic sequencing of the pedal skin (SG‐rich areas) and dorsal skin (SG‐free areas) of WT mice was performed, and GO enrichment analysis revealed that the SG microenvironment (WT pedal skin vs. dorsal skin up) was upregulated in response to hypoxia under physiological conditions (Figure 2A). Proteomic sequencing of the pedal skin and dorsal skin of 6w db/db mice revealed that the SG microenvironment of 6w db/db mice was also upregulated in response to hypoxia (Figure 2B). This provided molecular evidence of hypoxia due to inadequate vascular perfusion. This further confirmed that the sweating rates of normal subjects during normobaric hypoxia were significantly lower than those at sea level, suggesting that hypoxia has a direct effect on SG function, as evidenced by a reduced sweating rate [14].

Subsequently, based on the Gene Expression Omnibus (GEO) database (GSE80178), we found that DFU genes were upregulated in response to hypoxia relative to those in diabetic foot skin (DFS) and AW groups by GO enrichment analysis of the 129 genes overlapping between the DFU_vs_DFS up and DFU_vs_AW up groups (Figure 2C). The results of the HIF‐1α immunofluorescence staining of DFU and AW samples revealed substantial hypoxia in the DFU SGs (Figure 2D). This result provides further evidence for the association between progressive SG dysfunction and hypoxia.

Given that SGs are innervated by nerves and that sudomotor function is closely related to nerve function, it is necessary to investigate the genetic pathways altered in SG dysfunction due to the very early and advanced stages of DN [11]. Subsequent to the dissection of the sciatic nerve, SG function is completely lost (Figure S1). This investigation was based on the GEO database (GSE34889). The sciatic nerves of 24w db/db mice were upregulated in response to hypoxia relative to those of 8w db/db mice (Figure 2E middle panel), and peripheral nerve myelin formation and maintenance were downregulated (Figure 2E right panel). Similarly, compared with those of 24w db/m mice, the sciatic nerves of 24w db/db mice exhibited upregulated expression in response to hypoxia (Figure 2F middle panel), while downregulated expression was observed in terms mainly associated with “substance transport,” “axonogenesis, neurotransmitter transport, neuron development, and differentiation,” and “sensory perception” (Figure 2F right panel). In DN, reduced nerve conduction velocities correlate with the level of endoneurial hypoxia [15, 16], and disturbances in capillary blood flow can have an impact on the extraction of oxygen and glucose from the tissues [17]. While neurons require large amounts of energy to function properly, especially when propagating signals and releasing and reabsorbing neurotransmitters, a continuous supply of energy substrates to NC bodies and axons is required. The main energy substrates imported into the cell from the circulation are glucose and fatty acids, and neuronal uptake of these two substrates involves oxidative phosphorylation, a process that requires oxygen [18]. As the duration of diabetes increases, capillary dysfunction becomes more severe, exacerbating tissue hypoxia, which further exacerbates DN and affects neural function, manifesting as downregulation of genes related to neural function. The above results indicated that diabetic peripheral nerve impairment was associated with DM‐induced hypoxia, thereby offering novel insights into the pathogenesis of DN disorders. Therefore, it is necessary to investigate the impact of DM‐induced peripheral nerve impairment on SG function. The follow‐up study focused on the impact of nerves on SGs.

### 2.3 High‐Resolution Spatial Distribution of Neural Networks of SGs in Mice

**FIGURE 3:**
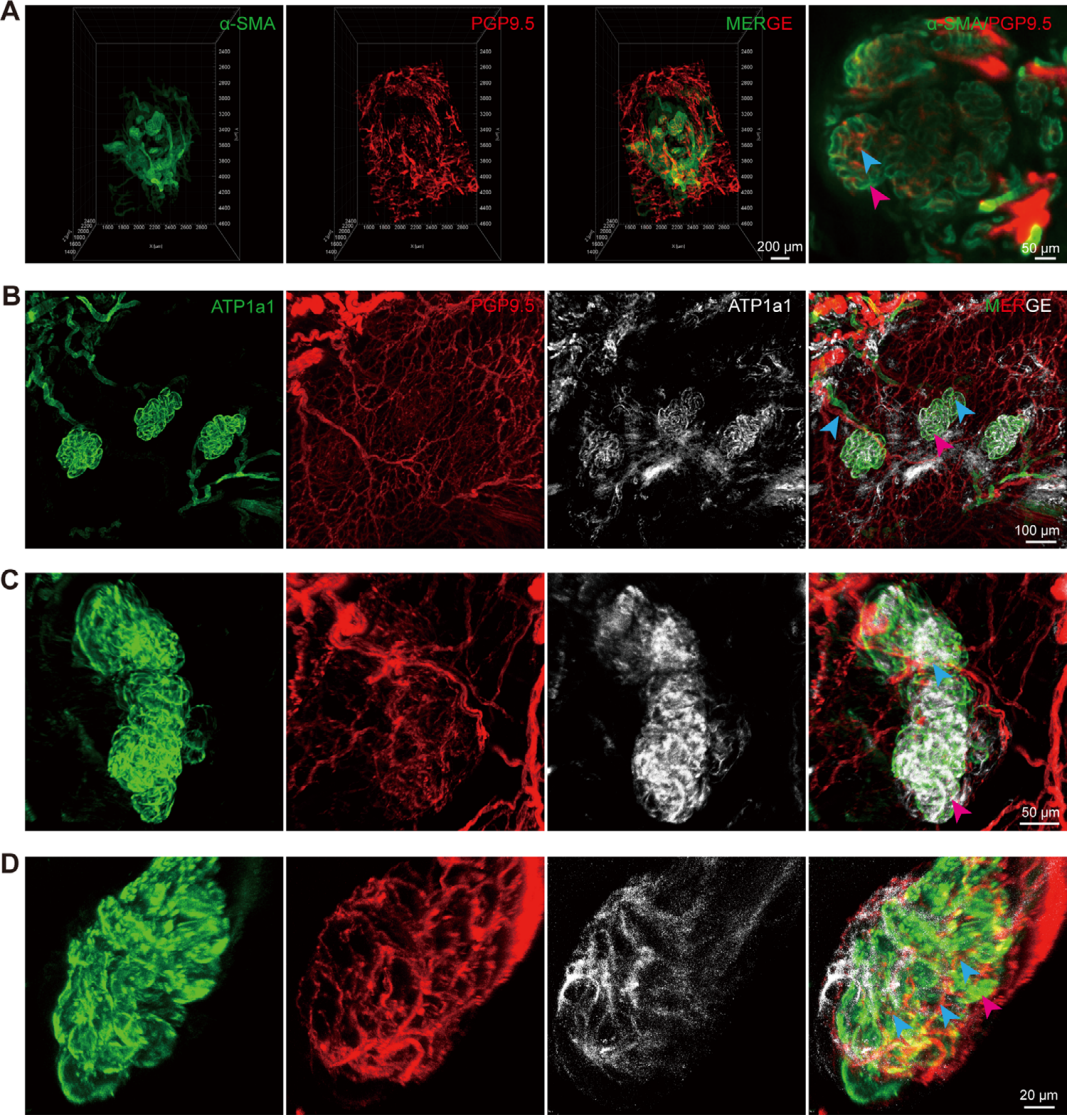
High‐resolution spatial distribution of neural networks of SGs in mice using a tissue clearing technique. (A) 3D reconstruction of the anatomical structure of the SG and adjacent neural network following tissue clearing using light‐sheet microscopy (scale bar: 200, 50 µm) and confocal microscopy (B–D) (scale bar: 100, 50, 20 µm). The pink and blue arrows indicate SGs and nerves, respectively.

Our previous studies demonstrated that SGs are surrounded by elaborate capillary networks, which facilitate SG function by providing a unique three‐dimensional (3D) vascular structure [5]. However, the anatomical relationship between the spatial distribution of SGs and nerves has not yet been elucidated. To map the spatial distribution of nerves in the footpad of mice and analyze the relationship between the location of nerves and SGs, 3D reconstruction of light‐sheet microscopy images of the paw pads of WT mice revealed the presence of a neural network in both the secretory and ductal portions of SGs. In particular, numerous nerves were distributed within the coiled sweat ducts, with the lateral branches exhibiting a high degree of connectivity, forming a sophisticated neural network that was closely arranged around the SGs (Figure 3A, Movie S1).

To achieve higher image contrast and resolution, Z‐stack scanning based on confocal microscopy was performed on the SG distribution region. SGs exhibited a close relationship with their adjacent neural networks, receiving multiple nerve fibers. These nerves divided into branches that extended longitudinally into the sweat ducts and wrapped extensively around the ducts, thereby facilitating better innervation of the SGs and sweat secretion (Figure 3B–D). This provided an anatomical basis for the potential influence of nerves on SG function.

### 2.4 Impaired SG Neurovascular Network in DFU Patients and DN Mice

**FIGURE 4:**
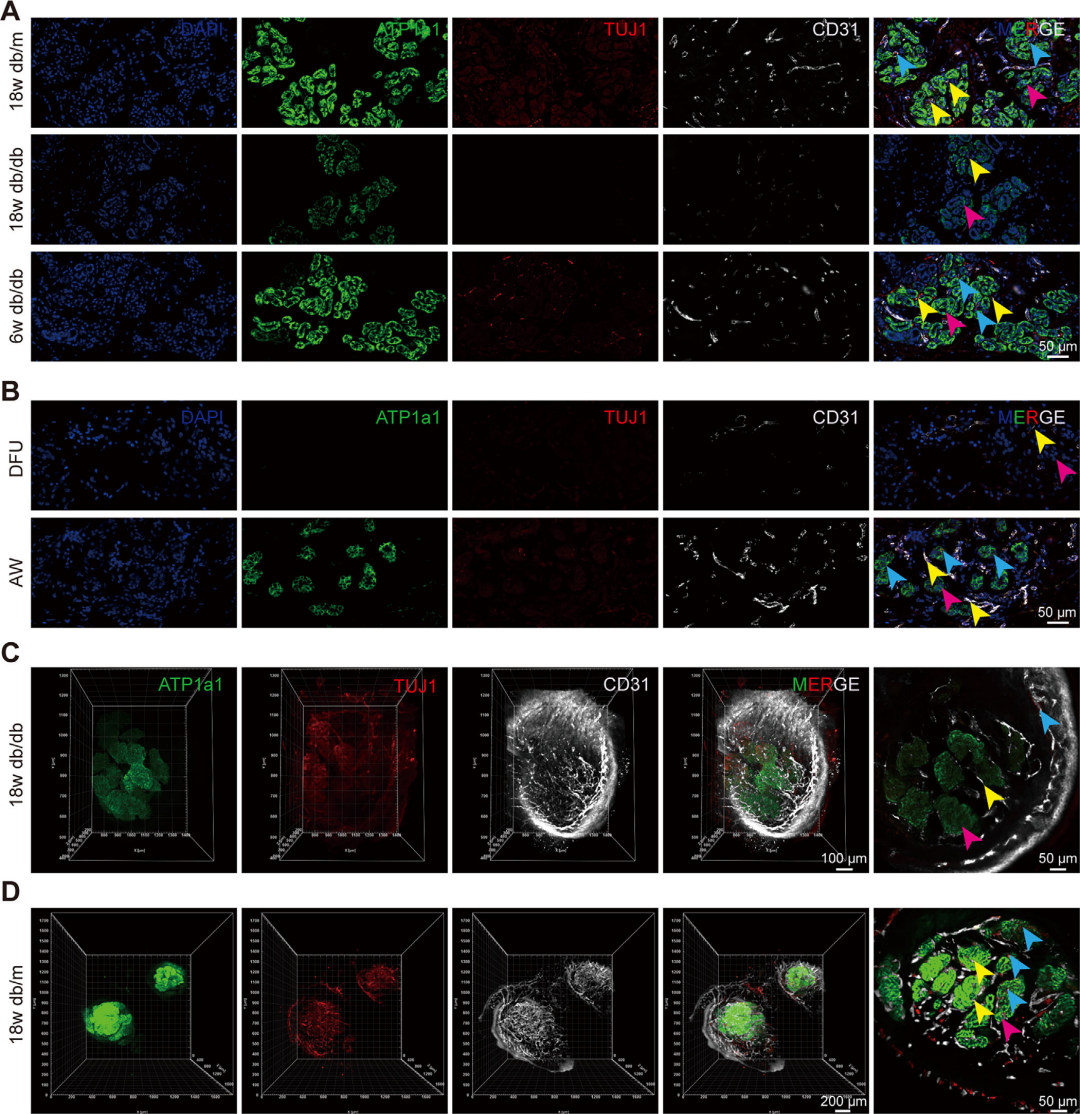
Histological staining and tissue clearing revealed an impaired neurovascular network around SGs. (A) Immunofluorescence histological sections showing the functional marker (ATP1a1), innervation (TUJ1) and vasculature (CD31) of SGs in 18w db/db, 18w db/m, and 6w db/db mice, and in patients with DFU and AW (B) (scale bar: 50 µm). (C, D) Light‐sheet microscopy images delineating the SGs and their neurovascular networks in 18w db/db and 18w db/m mice. 3D reconstruction of light‐sheet microscopy images was performed using Imaris software. Scale bar: 100, 50 µm (C); 200, 50 µm (D). The pink, yellow, and blue arrows indicate SGs, microvessels, and nerves, respectively.

Early and rapid reconstruction of the neurovascular network has been shown to exert a key influence on spinal cord injury repair [19], bone regeneration [20], wound healing [21, 22, 23], and recovery from brain trauma after stroke [24]. The vasculature plays a pivotal role in the formation of a reparative neurovascular niche, with the formation of neural structures following regeneration of the vascular bed [23, 24]. It is of paramount importance to facilitate the restoration of nerves to facilitate the functional recovery of tissues undergoing regeneration. Consequently, it can be conjectured that SG function is closely linked to its peripheral neurovascular network. To assess whether sudomotor dysfunction was associated with an impaired neurovascular network, immunofluorescence staining was performed, and the results revealed that ATP1a1, an SG functional marker, was sporadically expressed in the paw pads of 18w db/db mice compared with those of 18w db/m and 6w db/db mice. Furthermore, the number of nerves and blood vessels around the SGs was also significantly reduced (Figure 4A). In contrast, no significant differences were detected in the expression of K18, K19, or α‐SMA, which serve as SG structural markers [25] (Figure S2A). These findings were also corroborated in histological sections of skin samples from patients with DFU and AW patients (Figures 4B and S2B), where SG dysfunction was correlated with an impaired neurovascular network.

Tissue clearing further revealed a significant reduction in the number of nerves and blood vessels in 18w db/db mice relative to 18w db/m mice, accompanied by a decrease in the expression of a SG functional marker, suggesting an impaired neurovascular network around SGs in 18w db/db mice compared with that in 18w db/m mice. This finding reinforced the link between an impaired neurovascular network and SG dysfunction (Figure 4C,D).

### 2.5 Close Cellular Communication Between ECs, NCs, and SGCs

**FIGURE 5:**
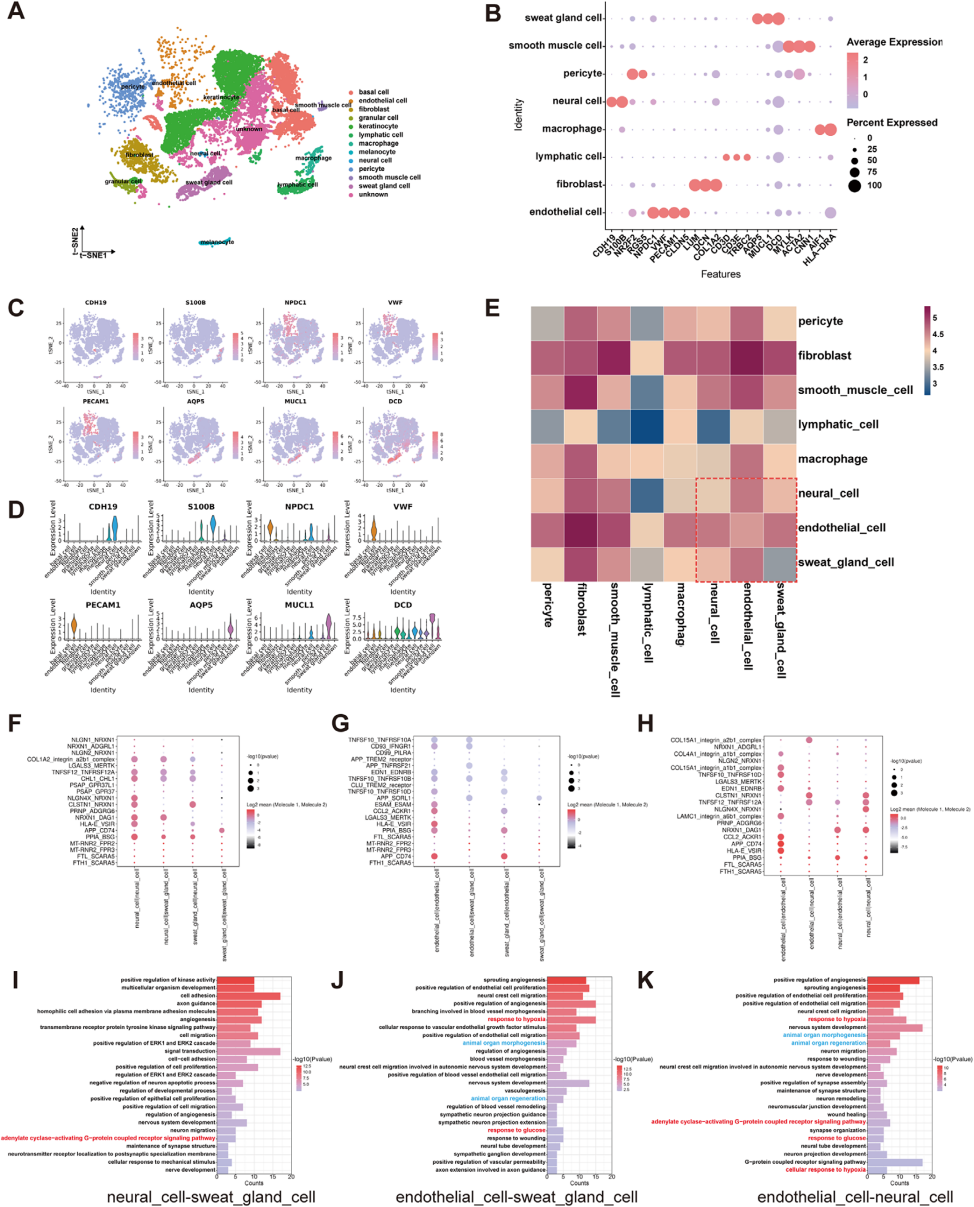
Close intercellular communication among neural cells (NCs), endothelial cells (ECs), and sweat gland cells (SGCs). (A) TSNE plot of normal skin cells, colored by cluster. (B) Dot plot showing the expression level and distribution of specific genes in different cell types present in the dermis of the skin. (C, D) Violin plots and TSNE data demonstrating the expression level and distribution of specific genes in NCs, ECs, and SGCs. (E) Heatmap illustrating interactions among cell types in the skin dermis obtained with CellPhoneDB. (F–H) Dot plot depicting the representative ligand–receptor pairs among NCs, ECs, and SGCs in normal skin. (I–K) GO enrichment analysis of receptor‐ligand genes between NCs and SGCs (I), ECs and SGCs (J), as well as ECs and NCs (K). *p* < 0.05 indicated significant enrichment.

Given the close anatomical relationship between SGs and their surrounding neurovascular network and the correlation between SG function and its neurovascular network, we hypothesized that there was an interaction between SGCs and their surrounding neurovascular network. To investigate the cellular interaction between NCs, ECs, and SGCs, the previous single‐cell RNA sequencing (scRNA‐seq) data [26], combined with the expression of signature genes for each cluster, were subjected to further analysis. This revealed that normal human skin could be classified into 12 cell types (Figure 5A). These cell types included NCs (identified by CDH19 and S100B), ECs (identified by NPDC1, VWF, PECAM1, and CLDN5), and SGCs (identified by AQP5, MUCL1, and DCD) (Figure 5B–D). To gain insight into the interactions between these three types of cells in their resident microenvironment, we revealed the cell–cell communication networks based on CellPhoneDB. The heatmaps demonstrated that there was close intercellular communication between ECs, NCs, and SGCs (Figure 5E). Subsequently, a bubble diagram was generated to visualize the receptor–ligand pairs between NCs, ECs, and SGCs (Figure 5F–H). To gain further insight into the biological function of the neural‐vascular‐SGC communication network, we performed GO enrichment analysis of ligand‒receptor genes between the three cell types based on the results of CellPhoneDB analysis (Figure 5I–K). The analysis revealed that the majority of the differentially expressed ligand‒receptor genes were primarily associated with blood vessels and nerves. These included genes involved in angiogenesis, blood vessel morphogenesis, axon guidance, nervous system development, neural crest cell migration, synaptic assembly, structural maintenance and organization, and response to pathological conditions, including response to hypoxia and glucose.

### 2.6 In Vitro SNIM Suggested Neural Support for SGC Function

**FIGURE 6:**
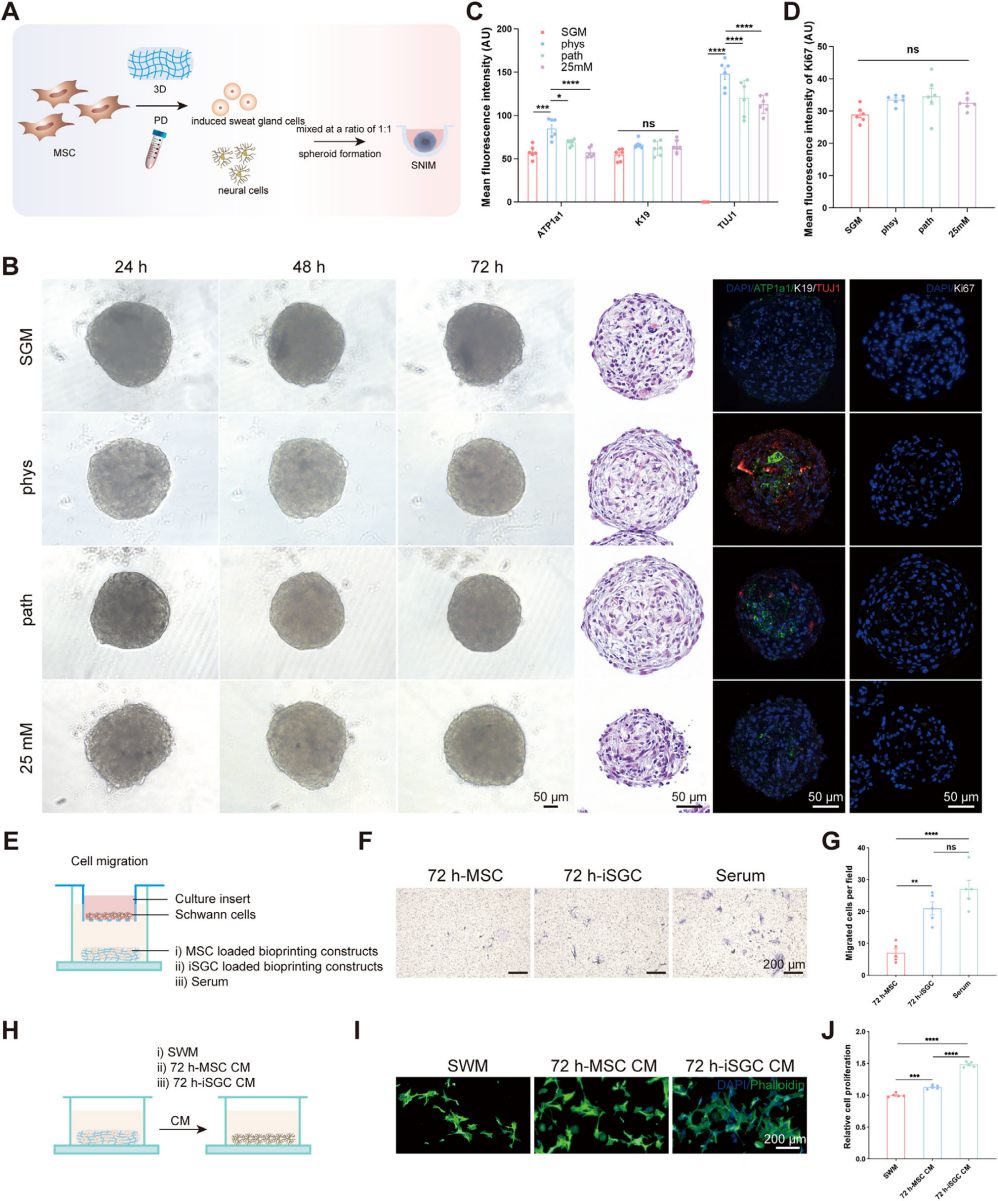
In vitro modeling of sweat gland cell (SGC)‐neural (NC) interactions and functional validation. (A) Schematic diagram of SGC lineage differentiation and the establishment of the SGC–NC interaction model (SNIM). (B) Morphology of SNIM under physiological and pathological conditions, corresponding HE and immunofluorescence staining of SG functional marker (ATP1a1), structural marker (K19), neural marker (TUJ1), and proliferation marker (Ki67) (Scale bar: 50 µm). (C) Statistical analysis of the mean fluorescence intensity of ATP1a1, K19 and TUJ1 in spheroids. *n* = 6 per group. (D) Quantification of the Ki67 expression level. *n* = 6 per group. (E) Schematic showing coculturing cell‐loaded bioprinting constructs and serum with Schwann cells. (F) Assessment of the migratory capacity of Schwann cells with different treatments by Transwell assay (scale bar: 200 µm). (G) Statistical analysis of migrated Schwann cells. *n* = 5 per group. (H) Schematic diagram of 72 h‐MSC and 72 h‐iSGC derived conditioned medium (CM) for Schwann cells. (I, J) Assessment of Schwann cell neurite growth and proliferation treated with 72h‐MSC‐ or 72h‐iSGC CM (Scale bar: 200 µm). *n* = 5 per group.

It has been shown that SGCs and ECs can interact with and without cell contact, as evidenced by the capacity of ECs to promote the aggregation of functional clusters of SGs, and the potent proangiogenic effects exhibited by SGCs [5]. Considering the inherent difficulties associated with the isolation, culture, and proliferation of primary SGCs, we successfully guided MSCs to differentiate specifically into induced sweat gland cells (iSGCs) using 3D bioprinting coupled with plantar dermis (PD) homogenates, which can provide structural (3D bioprinting) and biochemical cues (PD) imperative for functional SG lineage differentiation [27] (Figure S3A–C). Given the inability of SGCs to form a spheroid interaction model with HT22 or N2a cells, and the lack of proliferative capacity in primary DRG cells (Figure S4A–C), subsequent experiments were conducted using Schwann cells. Based on the close spatial location and interrelationship between SGs and neural networks, we successfully explored a direct contact spheroid model between SGCs and Schwann cells and explored changes in the SNIM under physiological and pathological conditions (Figure 6A,B). The addition of Schwann's medium did not affect the expression of neural markers or the proliferation of the spheroids (Figure S5A–C). Following supplementation with Schwann cells, ATP1a1 in the “phys” group was significantly greater than that in the “SGM” group. However, after the intervention of pathological factors (high glucose), ATP1a1 was significantly lower in the “path” and the “25 mM” groups than in the “phys” group. Notably, there was no significant difference in the expression of an SG structural marker (Cytokeratin 19, K19) or in proliferation (Ki67) among the different groups (Figure 6C,D), excluding structural damage or proliferation‐restricted interference. These results suggested that the establishment of this SNIM could be a valuable method for studying the interactions between SGCs and NCs under physiological and pathological conditions. The establishment of the SNIM validated the direct support of NCs on SGC function and the impact of elevated glucose levels on the interplay between SGCs and NCs occurred independently of the influence on cellular proliferation.

To substantiate the mutual interaction between SGCs and NCs, Transwell assays were conducted to evaluate the impact of iSGCs on Schwann cell migration (Figure 6E). Differentiated MSC induced by 3D culture was used as a control. The results showed that 72 h‐iSGC loaded bioprinting constructs enhanced the migration of Schwann cells (Figure 6F,G). As shown in the schematic diagram (Figure 6H), conditioned media (CM) derived from 72 h‐MSC and 72 h‐iSGC were used to coculture with the Schwann cells. The proliferation of Schwann cells was promoted by the CM of iSGC cultured for 72 h, as evidenced by the CCK‐8 assay (Figure 6J), as well as their neurite growth (Figure 6I). This finding indicates that SGCs can promote nerve regeneration via a paracrine pathway.

## 3 Discussion

Patients suffering from DM may experience a loss of thermoregulatory sensation, a condition attributable to the dysfunction of sensory nerve endings. Consequently, the central nervous system is unable to respond to increased temperature and transmit it to downstream signaling pathways, resulting in hypohidrosis. Autonomic neuropathy can also lead to hypohidrosis through the inhibition of the neurotransmitter release to the SGs by dysfunction of autonomic nerve terminals [28].

Our study provides novel insights into the mechanisms underlying sudomotor dysfunction, a significant yet understudied complication, in diabetic patients. In contrast to previous findings suggesting structural impairments in the SGs of diabetic patients [3, 4], we observed a paradoxical scenario in which the SGs appeared structurally normal but functionally impaired. Our discovery aligns with reports of no significant atrophy in eccrine SGs between anhidrotic and hyperhidrotic areas in diabetic patients [8]. These findings suggest a progressive deterioration in SG function preceding structural changes, highlighting a window for therapeutic interventions aimed at preserving SG function during disease progression. Our integrative multiomics analysis revealed upregulation of the SG microenvironment and peripheral nerves in response to hypoxia, underscoring adaptive responses to metabolic stress in diabetes. This finding aligns with previous studies indicating hypoxia‐induced changes in gene expression linked to angiogenesis and oxidative stress in diabetic complications. Importantly, our use of tissue optical clearing techniques advanced the visualization of the 3D neurovascular network around SGs in mice, surpassing previous two‐dimensional assessments [29]. Furthermore, histological staining and tissue clearing techniques unveiled impaired neurovascular networks surrounding SGs in DN model mice, providing a comprehensive view of the intricate relationships supporting SG function. scRNA‐seq analysis elucidated intercellular communication among NCs, ECs, and SGCs, revealing their involvement in angiogenesis and nerve development. Our analysis of differentially expressed receptor‒ligand genes highlighted their roles in blood vessel and gland development, as previously reported [5].

Chronic hyperglycemia‐induced diabetic polyangiopathy significantly impacts tissue oxygenation, exacerbated by inflammatory cell recruitment with high oxygen consumption, which disrupts the SG microenvironment and neurovascular network critical for sudomotor function. Our findings highlight that diabetic patients develop sudomotor dysfunction due to the upregulation of the SG microenvironment, DFU, and peripheral nerves in response to hypoxia. Interactions between ECs and SGCs, as well as between ECs and NCs, show responses to hypoxia and glucose (Figure 5I–K). Particularly noteworthy is that SGCs express G protein‐coupled receptors that mediate sweat secretion [28], and the receptor‒ligand genes between NCs and SGCs, and between ECs and NCs are involved in activating the adenylate cyclase‐activating G‐protein coupled receptor signaling pathway, crucial for sweat secretion (Figure 5I–K). These interactions may be disrupted in DM patients, contributing significantly to sudomotor dysfunction. This underscores the urgent need for targeted therapies addressing these cellular interactions. Our findings, together with those of previous studies [5], suggest reciprocal interactions between SGs and the vascular niche, and suggest that neural support plays a pivotal role in SG function. Moreover, SGs are among the most developed organs in humans, where interactions between ECs and SGCs, as well as between ECs and NCs, are crucial for the development and regeneration of glandular tissues (Figure 5J–K). These insights not only advance our understanding of diabetes‐induced sudomotor dysfunction but also emphasize the critical role of SG health in broader DM management strategies.

In addition, the early stages of DN often manifest without overt clinical signs, posing challenges for timely diagnosis. The gold standard diagnostic method, skin biopsy, is invasive and time‐consuming and requires specialized equipment and personnel, limiting its suitability for routine screening [30]. Moreover, it carries risks such as infection, pain, and bleeding [31]. Corneal confocal microscopy (CCM) offers a noninvasive and rapid in vivo assessment of sensory C‐fibers, yet its widespread adoption is hindered by high costs and the need for specialized equipment [31]. Early autonomic dysfunction is a hallmark of this condition, with SG dysfunction being among the earliest detectable abnormalities [32]. Sudomotor assessment has been widely acknowledged as a crucial strategy for the early detection of neuropathy in diabetic patients, providing indirect insight into the health of the unmyelinated autonomic nerve fibers innervating the SGs [30, 33, 34]. Several experimental sudomotor testing methods exist for detecting small fiber neuropathy, including quantitative sudomotor axon reflex tests (QSART), thermoregulatory sweat tests (TST), sympathetic skin response (SSR), Neuropad, Sudoscan, and quantitative direct and indirect axon reflex tests (QDIRT) [33]. Early detection of autonomic dysfunction can mitigate cardiovascular events and reduce the frequency and severity of complications such as retinopathy and nephropathy [35]. In addition, the DN stage independently predicts sudomotor dysfunction and SG duct size [3], offering insights into the feasibility of restoring SG function based on disease progression. Furthermore, the strong correlation between skin hydration and tissue oxygenation underscores their role as reliable predictors of wound healing potential. Enhancing wound tissue hydration is pivotal for promoting chronic wound healing, highlighting the significance of restoring SG function [36, 37].

Sweat also serves as a diagnostic biofluid, with its dynamic quantification and composition analysis offering valuable insights into physiological health, stress levels, nutritional status, and exposure to substances [38]. The noninvasive collection of sweat holds promises across various medical applications, including disease management, drug screening, and monitoring hydration in athletic performance.

However, our study has several limitations. The sample size of DFU patients and DN mice in our study was modest, potentially limiting the generalizability of our findings to broader populations. Furthermore, while our in vitro SNIM effectively simulated SGC–NC interactions, it does not fully replicate the complex in vivo environment, which could impact the clinical translation of our results. Despite these constraints, our findings provide valuable insights and suggest promising directions for future research. The predominant perspective is that diabetic sudomotor dysfunction is attributable to diabetic autonomic neuropathy. The majority of available treatments are symptomatic and are employed to enhance autonomic function [35]. There is an absence of effective therapeutic interventions for addressing diabetic sudomotor dysfunction. Future solutions to diabetic sudomotor dysfunction could focus on developing hypoxia‐targeted therapies to improve SG oxygenation and leveraging SGC–EC/NC interactions for neurovascular regeneration. To strengthen the validity of our conclusions and further explore therapeutic opportunities, further research is needed to validate these strategies through large‐scale clinical cohorts and advanced in vitro models, such as SG organoids. These efforts will be crucial in validating our findings and advancing the understanding and treatment of DN.

## 4 Conclusion

In summary, our study has illuminated the intricate mechanisms underlying sudomotor dysfunction in DM patients. We have identified significant findings including the upregulation of the SG microenvironment in response to hypoxia, the pivotal role of the neurovascular‐SG axis in DM, and the establishment of SNIM. These novel insights hold promise for shaping future therapeutic strategies aimed at improving clinical outcomes for diabetic patients. By establishing new scientific foundations and therapeutic targets, our findings contribute valuable groundwork for advancing the clinical management of diabetes.

## 5 Materials and Methods

### 5.1 Human Skin Samples

DFU skin tissues were collected through the debridement of nonhealing foot ulcer wounds (clinically confirmed need for debridement or amputation), which were referred to as the DFU group, while tissues from healthy patients were obtained during debridement following acute traumatic injury and were referred to as the healthy group. All tissues were excised from the margin of the wound and fixed in 4% formaldehyde (Solarbio, China). Patient characteristics are shown in Table S1.

### 5.2 Animals

The early stage of DN in db/db mice is considered to be 18 weeks [11]. Therefore, 18w db/db mice were used for the subsequent experiments. Newborn and 6w male mice (C57BL/6J), 6w and 18w male (BKS.Cg‐Lepr^db^, db/db) diabetic mice, and control mice (BKS‐Lepr, db/m) were purchased from Viewsolid (Beijing, China) Biotechnology Co. Ltd. and used for further study (male mice exhibited a more pronounced diabetic phenotype than female mice). All the experimental animals were maintained in a standard specific‐pathogen‐free laboratory environment with access to sterilized food and fresh water ad libitum. Anesthesia was induced with sodium pentobarbital or isoflurane during the experiments, and all animal experiments were conducted in concordance with the principles of animal welfare.

### 5.3 Sweat Test and Blood Flow Perfusion Assessment

Iodine/ethanol (2% w/v) was topically administered to the hind paw pads of the mice. After the iodine/ethanol solution completely dried, the starch/castor oil solution (1 g mL^−1^) was evenly and thickly applied to the hind paws of the mice. Subsequently, 20 µL of 100 mM acetylcholine solution (Sigma‒Aldrich, USA) was injected subcutaneously into the hind footpads of the mice. It is important to note that prolonged exposure of starch‐covered hind footpads to the atmosphere may result in undesirable color changes due to moisture [39]. Consequently, representative images were captured and documented after 5 min to ensure the stable emergence of dark sweating spots.

To assess the blood flow perfusion of the paw pads, a laser Doppler perfusion imaging system (Perimed, Sweden) was used. The overall mouse paw pad was identified as ROI1, while the SG region was designated ROI2. The perfusion signal is displayed in a color scale bar, with dark blue representing low perfusion and red representing high perfusion.

### 5.4 Proteomic Sequencing and Differentially Expressed Gene (DEG) Identification From GEO Datasets

The hind paw skin and dorsal skin of 6w wild‐type (WT) mice and 6w db/db mice were collected. Tandem mass tag (TMT)‐based quantitative proteomic sequencing was performed with the assistance of LC‐Bio Technology Co. Ltd. A *p* value < 0.05 was considered to indicate statistical significance.

DEGs identified from GEO datasets were downloaded from the GEO database using GEO2R. Genes with a fold change > 2.0 and a *p* value < 0.05 were considered significant. Gene Ontology (GO) enrichment analysis was conducted using DAVID [40, 41].

### 5.5 Cell Culture, Isolation, and Plantar Dermis Homogenates Preparation

Primary Schwann cells and primary dorsal root ganglia (DRG) cells were purchased from Zhejiang Meisen Cell Technology Co. Ltd. (Hangzhou, China) and maintained in primary neuronal culture systems (Meisen, China) and primary Schwann cell culture systems (Schwann's medium, Meisen, China). The HT22 and N2a cell lines were obtained from the Chinese Academy of Sciences Cell Bank (Shanghai, China) and maintained in DMEM (Gibco, USA) supplemented with 10% fetal bovine serum (FBS, Gibco, USA) at 37°C with 5% CO_2_.

Primary bone marrow‐derived mesenchymal stem cells (BMSCs) were isolated and cultured in accordance with previously reported methods [42]. In brief, the clean femurs and tibial fibulae of neonatal mice that had been euthanized and soaked in 75% alcohol for 30 min were cut into pieces and then incubated in complete MesenCult proliferation culture medium (Mouse, STEMCELL, Canada). Cells at passages 2–4 were used. The subsequent BMSCs are denoted as MSCs.

PD homogenates were prepared based on our previous work [42]. The neonatal mouse paws were separated using microscissors and placed in a sterile grinder. Sterile phosphate buffered saline (PBS) was added to the grinder, and the samples were fixed to 5 mL of PBS/g of tissue, which was fully ground to form a tissue homogenate. Subsequently, the supernatant was subjected to centrifugation at 12,000 × g for 20 min at 4°C. Next, the supernatant was filtered through a 0.22 µm filter and transferred to a new Eppendorf tube for further experiments or storage at −80°C.

### 5.6 SGC Lineage Differentiation

Given the pivotal role of structural cues in the differentiation of MSCs toward iSGCs, bioprinting and SG induction were based on our previous study [5, 42]. Briefly, the bioink was formulated with 1% (w/v) sodium alginate (Sigma‒Aldrich, USA) and 3% (w/v) gelatin (Sigma‒Aldrich, USA). MSCs were resuspended in a 1:1 mixture of 1 mL of PD and MSC medium at a cell density of 1×10^7^/mL and then gently mixed with 9 mL of prefabricated ink to form 10 mL of bioink. Subsequently, the cell‐containing bioink was continuously bioprinted using an extrusion‐based 3D bioprinter (Regenovo, China) and stored at 4°C for 30 min to maintain the shape of the constructs. The bioprinted constructs were then crosslinked with calcium chloride (2.5%, w/v) for 3 min and washed twice with deionized water. Next, the crosslinked cell–containing constructs were cultured in SG medium (SGM) or SGM containing 5% PD at 37°C in a 5% CO₂ standard incubator for 7 days, with the medium exchanged every other day. The group with PD added was designated the “iSGC” group, and the group without PD was named the “MSC” group.

Specifically, the SGM contained DMEM/F12 (HyClone, USA) basal culture medium and a list of supplements, including 3,3′,5‐triiodo‐L‐thyronine (2 ng/mL, Sigma‒Aldrich), hydrocortisone hemisuccinate (400 µg/mL, Sigma‒Aldrich), epidermal growth factor (10 ng/mL, PeproTech, USA), 1% (v/v) insulin‐transferrin‐selenium (Gibco), 5% (v/v) FBS (Gibco), and 1% (v/v) penicillin–streptomycin solutions.

### 5.7 Histological and Immunological Staining

The paw pads of the mice were fixed in 4% formaldehyde for 24 h and embedded in paraffin according to standard procedures. The embedded tissues were sectioned at a thickness of 4 µm, followed by histological studies. Hematoxylin and eosin (H&E) staining was then conducted according to routine histological protocols to visualize the anatomical structure of the samples.

The 3D bioprinted constructs, which had been cultured for 7 days, were subjected to a series of processes, including fixation, dissolution, centrifugation, resuspension, and transfer onto adhesive slides. The samples were then dried at 60°C for 30 min and subjected to subsequent immunofluorescence staining. Cells from the “MSC” and “iSGC” groups were isolated on day 7 of differentiation and stained with SG‐specific biomarkers to confirm differentiation efficacy.

The aforementioned sections were stained with tissue‐specific markers according to standard immunological staining protocols. Following antigen retrieval and blocking, the sections were incubated with the following primary antibodies overnight: anti‐cytokeratin 14 (1:300, ab7800, Abcam), anti‐cytokeratin18 (1:300, ab668, Abcam), anti‐cytokeratin19 (1:300, ab52625, Abcam), anti‐ATP1a1 (1:300, ab7671, Abcam), anti‐HIF‐1 alpha (HIF‐1α) (1:500, ab179483, Abcam), anti‐alpha smooth muscle actin (α‐SMA) (1:1000, ab7817, Abcam), anti‐beta III tubulin (TUJ1, 1:100, 801202, Biolegend), anti‐PGP9.5 (1:500, ab108986, Abcam), anti‐CD31 (1:1000, ab281583, Abcam), and anti‐Ki67 (1:200, ab16667, Abcam). Subsequently, the sections were incubated with secondary antibodies, including Goat anti‐Rabbit IgG (H+L) Cross‐Adsorbed Secondary Antibody, Alexa Fluor 647 (1:500, A‐21245, Invitrogen); Goat anti‐Mouse IgG2a Cross‐Adsorbed Secondary Antibody, Alexa Fluor 568 (1:500, A‐21134, Invitrogen); Goat anti‐Mouse IgG1 Cross‐Adsorbed Secondary Antibody, Alexa Fluor 488 (1:500, A‐21121, Invitrogen); Goat Anti‐Mouse IgG H&L, Alexa Fluor 594 (1:500, ab150116, Abcam); and Goat Anti‐Rabbit IgG H&L, Alexa Fluor 488 (1:500, ab150077, Abcam), for 2 h at room temperature. For the assessment of neurite growth, cells were stained with FITC Phalloidin (CA1620, Solarbio, 1:200) for 30 min. Finally, 4’,6‐diamidino‐2‐phenylindole (DAPI) Fluoromount‐G (0100‐20, SouthernBiotech) was used to stain the nucleus. Images were captured with a confocal microscope (Leica, SP8 FALCON).

### 5.8 Tissue Clearing

According to the manufacturer's protocol [43], the footpads of mice that had undergone cardiac perfusion were isolated, fixed in 4% formaldehyde (Solarbio, China), and rendered transparent using an FDISCO kit (JARVIS, China). Following transparency, the samples were scanned and imaged in three dimensions using a LiTone XL light sheet illumination microscopic imaging system. 3D reconstruction images and movies were fabricated using Imaris 9.7 (Bitplane, Switzerland). 3D images were generated using the “snapshot” function, while the movies were generated using the “animation” function.

### 5.9 scRNA‐Seq Data Processing and Analysis

The scRNA‐seq data for this study were derived from our previous study [26]. t‐Distributed stochastic neighbor embedding (t‐SNE) was employed to visualize the data. Subsequently, 12 cell clusters were identified and characterized based on the Cell Marker database and published literature. Further analysis of the intercellular communication of cells in the dermis was conducted, and GO enrichment was performed on the receptor‒ligand pairs of ECs, NCs, and SGCs. GO enrichment analysis was conducted using DAVID [40, 41].

### 5.10 RNA Extraction and Quantitative Real‐Time PCR

Total RNA was harvested using TRIzol reagents (Invitrogen, USA) according to the manufacturer's instructions. The RNA was then converted to complementary DNA (cDNA) using a PrimeScript RT reagent Kit with gDNA Eraser (Takara, China). Quantitative analysis of gene expression was performed using TB Green Premix Ex Taq II (Takara, China) on a QuantStudio 5 system (Applied Biosystems, USA). The 2^−∆∆CT^ methods were used and gene expression was normalized to the expression of Gaphd. The primers used are shown in Table S2.

### 5.11 SNIM Establishment

In light of our previous research, we developed a direct contact spheroid model to study the interactions between SGCs and NCs in vitro [26]. In summary, SGCs dissolved from 3D bioprinted constructs and NCs (HT22, N2a, Schwann, and DRG) were mixed at a ratio of 1:1, respectively. The cell density was then adjusted to 1.5 × 10^4^ cells/mL, and 3000 cells (200 µL) were transferred to 96‐well ultra‐low adherence plates (Labselect, China) for 72 h incubation. The morphology of spheroids was regularly recorded by phase contrast microscopy (Leica, DMI4000B).

The specific groupings of SNIM were as follows: the “SGM” group consisted of spheroids formed by culturing iSGCs with SGM alone. In the “SGM+SWM” group, spheroids were formed by culturing iSGCs with a 1:1 mixture of SGM and primary Schwann cell culture systems (Schwann's medium, SWM) to exclude the effect of SWM on the expression of a neural marker (TUJ1) in the spheroids of the subsequent group. The “phys” group consisted of spheroids formed by culturing iSGCs and Schwann cells with a 1:1 mixture of SGM and SWM. The “path” group consisted of spheroids formed by mixing iSGCs and Schwann cells with a 1:1 mixture of SGM and SWM, with the addition of 45 mM glucose. The “25 mM” group consisted of spheroids formed by mixing iSGCs and Schwann cells that had been cultured after 45 days of pretreatment with an additional 25 mM glucose in SWM and with a 1:1 mixture of SGM and SWM.

### 5.12 Transwell Assay

Transwell assays were conducted to evaluate the impact of iSGCs on Schwann cell migration. The bioprinting constructs loaded with MSC, iSGC, and serum were positioned in the lower chamber of the Transwell unit and cultured for 72 h. Thereafter, the Schwann cells were seeded in the upper chamber for a further 36 h. The Schwann cells were then fixed with 4% paraformaldehyde. Following a thorough rinse with PBS thrice, the cells were stained with 0.1% crystal violet (Solarbio, China) for 30 min. The non‐migrated cells in the upper chamber were then removed using cotton swabs, and five fields were randomly selected for quantification of the migrated Schwann cells.

### 5.13 Cell Proliferation Assessment

Schwann cell proliferation was assessed with cell counting kit‐8 (CCK‐8; Dojindo) assay. The cells were seeded in 96‐well plates at a density of 10^4^ cells per well, and different treatments were applied after cell adhesion. Following 36 h of intervention, the cells were incubated with CCK‐8 reagent for 1 h at 37°C. Cell proliferation was subsequently measured using a SPARK 10 M plate reader (TECAN).

### 5.14 Statistical Analysis

All data in this study are presented as the means ± SEMs of at least three independent experiments. Two‐group comparisons were performed by unpaired two‐tailed *t*‐tests, and comparisons between multiple groups were made by two‐way analysis of variance (ANOVA) with Tukey's multiple comparisons test. A *p* value < 0.05 was considered to indicate statistical significance. All the statistical analyses were conducted using GraphPad Prism 10.1.2 statistical software (GraphPad, USA). Significance is designated as follows: *p < 0.05; **p < 0.01; ***p < 0.001; ****p < 0.0001; ns, not significant.

## Author Contributions

X.F. and S.H. designed and directed the project. X.G. performed the experiments. X.G., Y.W., D.Z., and Z.Z. analyzed the data. Y.W., Y.T., Z.L., W.S., and Y.K. provided guidance and advice. All authors discussed the results and contributed to the final manuscript. X.G. and C.Z. wrote the manuscript. S.H. revised the manuscript. All authors have read and approved the final manuscript.

## Ethics Statement

Each patient provided informed consent prior to the commencement of the study. All procedures involving human subjects were approved by the Medical Ethics Committee of the Chinese PLA General Hospital and were performed in accordance with the Declaration of Helsinki principles (S2021‐337‐01).

The procedures involving experimental animals were approved by the Institutional Animal Care and Use Committee of Chinese PLA General Hospital (S2020‐407‐01).

## Conflicts of Interest

The authors declare no conflicts of interest.

## Supporting information



Supporting Information

Supporting Information

Supporting Information

## Data Availability

The raw data described in this paper are available from corresponding author upon reasonable request.
