# Supplementary material for: Hypoxia‐Driven Neurovascular Impairment Underlies Structural‐Functional Dissociation in Diabetic Sudomotor Dysfunction
Source: MedComm (2020). 2025 Apr 24;6(5):e70173. doi: 10.1002/mco2.70173 (PMC12019874; doi:10.1002/mco2.70173)
Supplement: Supplementary file 1 — Supporting Information [file MCO2-6-e70173-s001.docx]

**Table S1. Patient characteristics and sample information**

| Sample | Age | Gender (M/F) | Ulcer duration/location |
| --- | --- | --- | --- |
| DFU | 70 | F | 1 M |
| DFU | 68 | M | 3 M |
| DFU | 74 | F | 4 M |
| AW | 20 | M | Forearm |
| AW | 13 | F | Elbow |
| AW | 20 | F | Hand |

DFU, diabetic foot ulcer; AW, acute wound

Table S2. Primers of target genes for Real-time qPCR

| **Primers** | **Sequences (5’ to 3’)** |
| --- | --- |
| Gapdh-F | AGGTCGGTGTGAACGGATTTG |
| Gapdh-R | TGTAGACCATGTAGTTGAGGTCA |
| ATP1a1-F | GGGGTTGGACGAGACAAGTAT |
| ATP1a1-R | CGGCTCAAATCTGTTCCGTAT |
