# Supplementary material for: Hypoxia‐Driven Neurovascular Impairment Underlies Structural‐Functional Dissociation in Diabetic Sudomotor Dysfunction
Source: MedComm (2020). 2025 Apr 24;6(5):e70173. doi: 10.1002/mco2.70173 (PMC12019874; doi:10.1002/mco2.70173)
Supplement: Supplementary file 2 — Supporting Information [file MCO2-6-e70173-s003.docx]

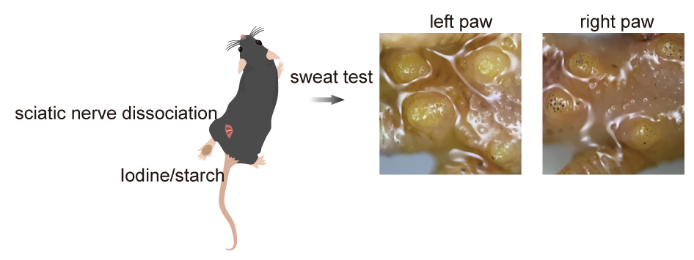


**Figure S1** Schematic diagram of the effect of sciatic nerve dissection on sweat gland function.


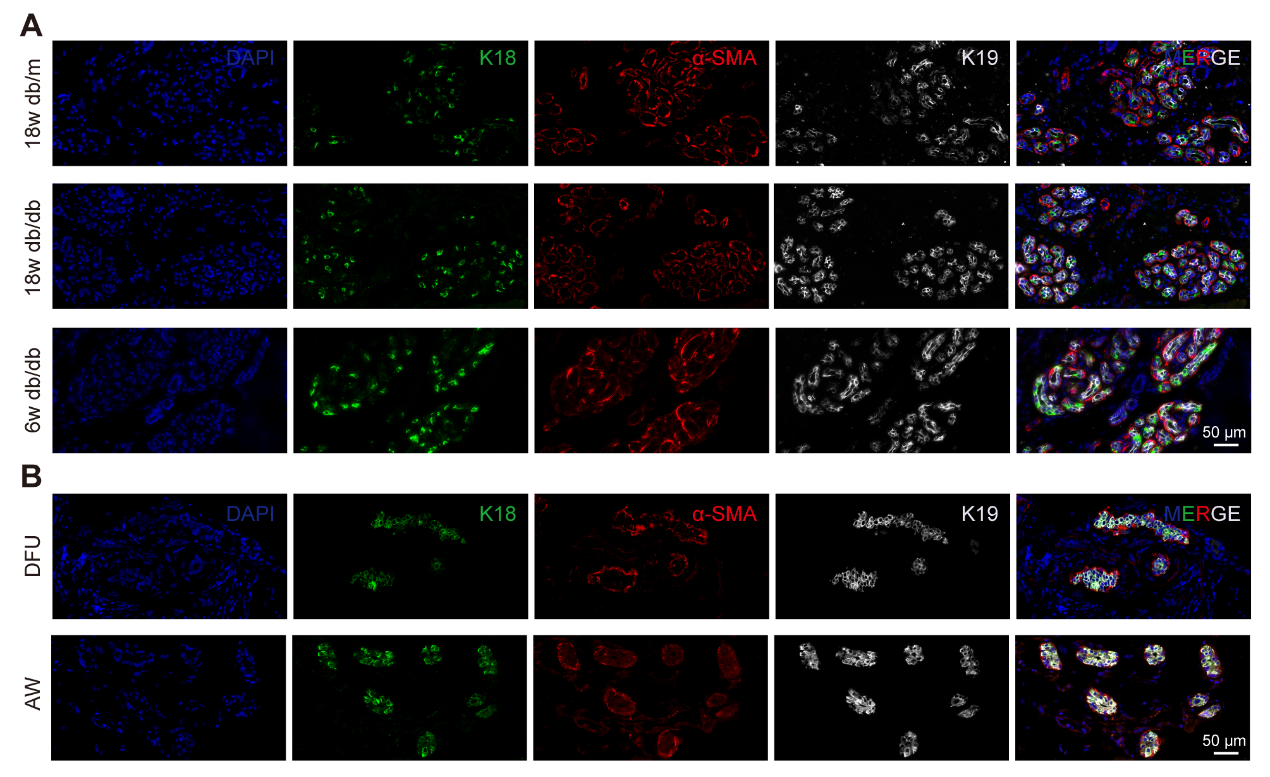


**Figure S2** Immunofluorescence histological sections showing the sweat gland structural markers of in 18w db/db, 18w db/m and 6w db/db mice (A), and in patients with DFU and AW (B) (scale bar: 50 μm).


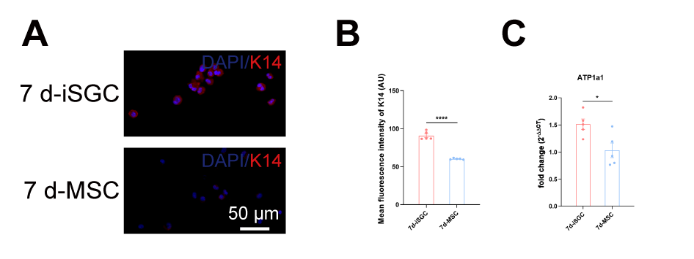


**Figure S3** Characterization of MSCs derived iSGCs by SG specific markers using immunofluorescence staining and statistical analysis (A, B) (scale bar: 50 μm), and RT-qPCR (C). n=5 per group.


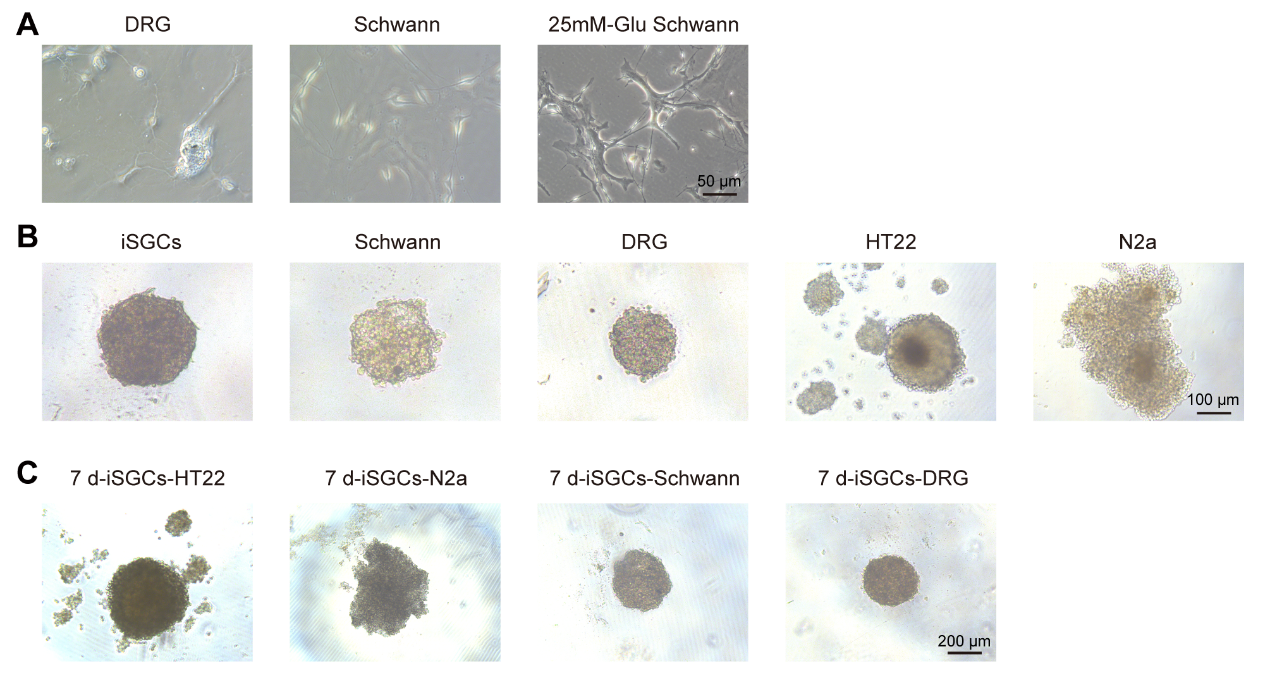


**Figure S4** Characterization of direct spheroid interaction models between sweat gland cells (SGCs) and four neural cells. (A) The light microscopic images of primary DRG, Schwann and 25mM glucose pretreated Schwann cells (scale bar: 50 μm). (B) Autonomous spheroid formation assessment of SGCs and four neural cells (scale bar: 100 μm). (C) Spheroid formation assessment between SGCs and four neural cells (scale bar: 200 μm).


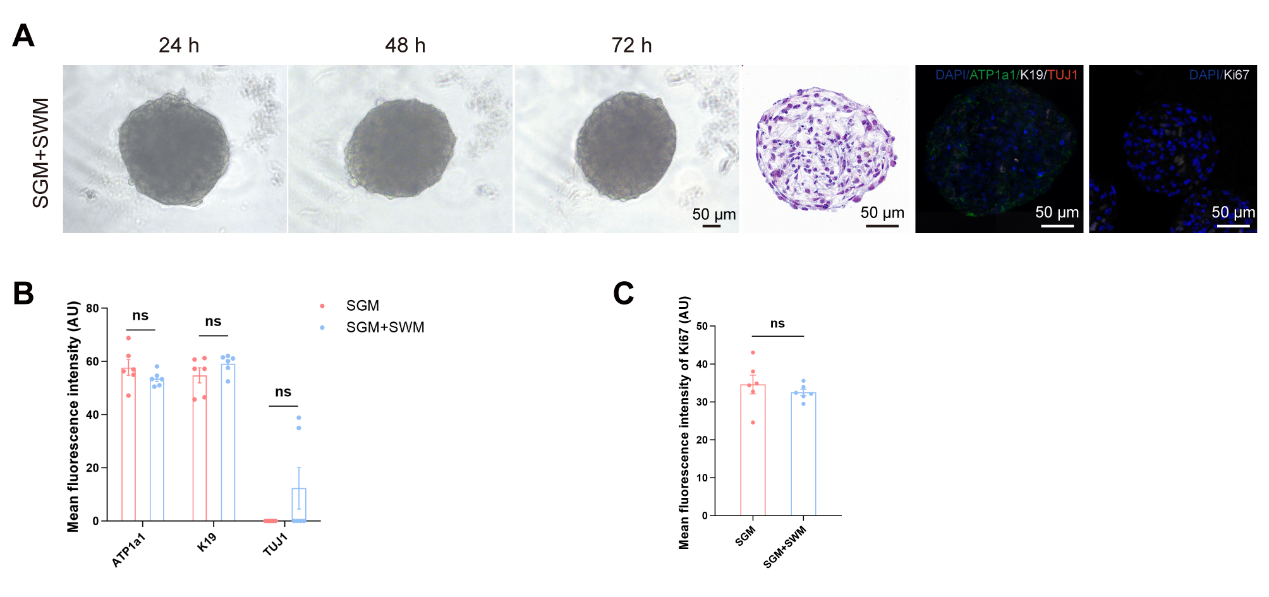


**Figure S5** Effects of the addition of Schwann’s medium on the expression markers of spheroids formed by SGCs. (A) Morphology, corresponding HE and immunofluorescence staining of spheroids formed by SGCs following the addition of Schwann’s medium (scale bar: 50 μm). (B) Analysis of the mean fluorescence intensity of SG functional marker, structural marker and neural marker, and (C) Ki67 in spheroids between “SWM” group and “SGM+SWM” group. n=6 per group.
